# Functional Properties of Protein Hydrolysates on Growth, Digestive Enzyme Activities, Protein Metabolism, and Intestinal Health of Larval Largemouth Bass (*Micropterus salmoides*)

**DOI:** 10.3389/fimmu.2022.913024

**Published:** 2022-07-19

**Authors:** Zhengyu Sheng, Giovanni M. Turchini, Jianming Xu, Zishuo Fang, Naisong Chen, Ruitao Xie, Haitao Zhang, Songlin Li

**Affiliations:** ^1^ Research Centre of the Ministry of Agriculture and Rural Affairs on Environmental Ecology and Fish Nutrition, Shanghai Ocean University, Shanghai, China; ^2^ School of Agriculture and Food, Faculty of Veterinary and Agricultural Sciences, The University of Melbourne, Melbourne, VIC, Australia; ^3^ Key Laboratory of Aquatic, Livestock and Poultry Feed Science and Technology in South China, Ministry of Agriculture and Rural Affairs, Zhangjiang, China

**Keywords:** protein hydrolysates, larval fish, protein metabolism, intestinal development, intestinal microbiota

## Abstract

The present study was conducted to investigate the effects of dietary inclusion of protein hydrolysates on growth performance, digestive enzyme activities, protein metabolism, and intestinal health in larval largemouth bass (*Micropterus salmoides*). The experimental feeding trial presented in this study was based on five isonitrogenous and isolipidic diets formulated with graded inclusion levels of protein hydrolysates, and it showed that protein hydrolysates improved growth performance, reduced larval deformity rate, and increased the activity of digestive enzymes, including pepsin and trypsin. Gene expression results revealed that the supplementation of protein hydrolysates upregulated the expression of intestinal amino acid transporters LAT2 and peptide transporter 2 (PepT2), as well as the amino acid transporters LAT1 in muscle. Dietary provision of protein hydrolysates activated the target of rapamycin (TOR) pathway including the up-regulation of TOR and AKT1, and down-regulation of 4EBP1. Additionally, the expression of genes involved in the amino acids response (AAR) pathway, ATF4 and REDD1, were inhibited. Protein hydrolysates inhibited the transcription of some pro-inflammatory cytokines, including IL-8 and 5-LOX, but promoted the expression of anti-inflammatory cytokines TGF-β and IL-10. The 16S rRNA analysis, using V3-V4 region, indicated that dietary protein hydrolysates supplementation reduced the diversity of the intestine microbial community, increased the enrichment of *Plesiomonas* and reduced the enrichment of *Staphylococcus* at the genus level. In summary, protein hydrolysates have been shown to be an active and useful supplement to positively complement other protein sources in the diets for largemouth bass larvae, and this study provided novel insights on the beneficial roles and possible mechanisms of action of dietary protein hydrolysates in improving the overall performance of fish larvae.

## 1 Introduction

Most fish larvae are not fully developed at hatching, and typically possess an immature digestive tract (1, 2), resulting in limited digestive capacity. Therefore, pre-digested protein, or protein hydrolysates, which are rich in peptides and free amino acids (AAs), have been suggested to be advantageous for larval feeding, as they should be more effectively absorbed compared to intact proteins (2, 3). Consistently, available evidence suggested that appropriate dietary protein hydrolysates content can improve absorption capacity, digestive tract development, intestinal health, growth performance and survival rate of some fish larvae (4–7). However, the underlying mechanisms accounting for these beneficial roles of protein hydrolysates remain unclear.

After ingestion, dietary proteins are broken down during digestion into free AAs, dipeptides or tripeptides, and then absorbed through peptide transporters or AA transporters, such as oligopeptide transporter (PepT), L-type amino acid transporter 1 (LAT1), and L-type amino acid transporter 2 (LAT2) (8, 9). In mammals, it has been well established that once a flux of AAs is absorbed and available to the organism, and this is then sensed by the target of rapamycin (TOR) pathway and the amino acid response (AAR) pathway, which both serve as major cellular trophic-sensing and signaling pathways regulating protein synthesis and downstream metabolism (10). The activation of TOR pathway, stimulated by the availability of AAs, regulates the growth of the organism by promoting protein synthesis and inhibiting autophagy (11); whereas the AAR pathway, triggered by an imbalanced AAs supply, has the opposite effect and inhibits global protein synthesis (12). Consistently, in teleosts, free AAs absorbed from dietary protein are transported to, and utilized by, muscle for protein synthesis (13), and the TOR and AAR signaling pathways have also been shown to be involved in the regulation of protein metabolism in fish species (14–17). The inclusion of marine protein hydrolysates, and therefore providing a typical marine AAs composition, has been suggested to activate the TOR pathway, and to inhibit the AAR pathway in juvenile largemouth bass (*Micropterus salmoides*) (18); two pathways modifications that have been suggested to contribute to improving growth performance (16–18). However, little additional information is yet available on the actual effects and mechanisms of dietary protein hydrolysates inclusion on protein metabolism in fish larvae. Therefore, illustrating the influence of the inclusion level of dietary protein hydrolysates on TOR and AAR pathways, which should contribute to determine the variation in growth performance, is an important topic requiring further clarifications.

Growing evidences confirmed the potential roles of protein hydrolysates in promoting digestive tract development and maturation (19, 20), but little is known about the underlying mechanisms. Meanwhile, the diversity and abundance of intestinal microbiota is suggested to be related to the development of the digestion and mucosal system in fish larvae (21, 22). Previous studies have demonstrated that dietary protein hydrolysates inclusion significantly affected the intestinal microbiota of fish (20, 23). The inclusion level of 5% sardine protein hydrolysates caused an increased amount of intestinal lactic acid bacteria in South American catfish (*Rhamdia quelen*) (24), whereas an artificial diet with up to 50% fish protein hydrolysate was needed to promote the abundance of lactic acid bacteria in Persian sturgeon (*Acipenser persicus*) larvae (5). The above studies suggest that the influence of protein hydrolysates on larval intestinal microbiota is likely dose dependent and species-specific. Additionally, dietary inclusion of protein hydrolysate at appropriate levels has been well-demonstrated to improve the immune response and disease resistance of some teleosts, including juvenile barramundi, *Lates calcarifer* (25–27), Japanese sea bass, *Lateolabrax japonicus* (28) and red seabream (*Pagrus major*) (29), and larval European sea bass (*Dicentrarchus labrax*) (30). Largemouth bass is a commercially important species, and it is widely cultured in China where its production is rapidly growing and has exceeded 619 thousand tons in 2020 (31). However, despite the size of the industry, the culture of larval largemouth bass is still mainly relying on the use of live feed; a practice that has some substantial disadvantages, such as high prices and instable supply, it is labor intensive, it brings risks of carrying pathogenic microorganisms and it might provide unbalanced nutrition, resulting in high larval mortality rates. However, fish larvae are not able to effectively utilize currently available compounded feed, and this is believed to be so, mainly because of their immature digestive tract.

Considering all of the above, the present study was conceived and then conducted to investigate the effects of dietary protein hydrolysates on growth performance, digestive enzymes activity, protein metabolism, and intestinal health of larval largemouth bass. It was envisaged that the findings of this study would contribute to current knowledge of larval fish nutrition, and would provide additional scientific understanding needed for the formulation and realization of effective artificial micro-diet, and therefore facilitating a more viable growth of the sector.

## 2 Materials and Methods

### 2.1 Experimental Diets

The main protein ingredients, white fish meal, fermented soybean meal, shrimp meal and blood meal, were blended and pre-mixed (**Supplementary Table 1**), and then were hydrolyzed with 2% neutral protease (50,000 U/g, Youtell Biochemical Co., Ltd., Hunan, China) and 1% keratinase (600,000 U/g, Novus International Trading Co., Ltd., Shanghai, China) in a 50°C water bath for 3h. After the enzymatic treatment, the hydrolysate was kept at 105°C for 30 min to sterilize and render the enzyme inactive, then dry for 15 hours at 80°C. The molecular weights of the protein hydrolysates peptide molecular weight fractions (**Supplementary Table 2**) were analyzed in the Analysis and Testing Center of Jiangnan University (Wuxi, China), following standard procedures, whereas the contents of free amino acids in protein hydrolysates (**Supplementary Table 3**) were analyzed by a commercial laboratory (Xi’an United Nations Quality Detection Technology Co., Ltd). The protein hydrolysis degree of pre-treated protein ingredients was 40.94%, which was measured with the trichloroacetic acid (TCA) method (32).

Five isonitrogenous (crude protein, 59%) and isolipidic (crude lipid, 13%) diets were formulated as follows. The control diet was produced using un-hydrolyzed pre-mixed protein as the main protein source (PH0). The other four diets were formulated with protein hydrolysates replacing approximately 25%, 50%, 75%, 100% the un-hydrolyzed protein pre-mixture, and were named PH25, PH50, PH75, PH100, respectively (**Table 1**). The amino acids compositions of experimental diets are presented in **Table 2**. Diets were manufactured following previously described procedures (33), and then the dried diets were ground to micro-diets with particle size ranged from 0.18 mm to 0.83 mm. The feeding management procedures for the use of experimental diets with different size was implemented according to the procedures previously described by Wang et al. (34). All diets were packed in double sealed plastic bags, and stored at -20°C until use.

**Table 1:** Formulation and chemical composition of experimental diets (% dry matter).

Ingredients	PH (%)				
	0	25	50	75	100
Pre-mixed protein ingredients^a^	70.73	53.05	35.36	17.68	0.00
Protein hydrolysates^b^	0.00	18.25	36.5	54.75	73.00
Fish protein powder^c^	4.00	4.00	4.00	4.00	4.00
Wheat gluten meal^d^	5.00	5.00	5.00	5.00	5.00
Sodium caseinate^d^	3.00	3.00	3.00	3.00	3.00
Yeast hydrolysate^d^	0.50	0.50	0.50	0.50	0.50
Starch^d^	3.00	3.00	3.00	3.00	3.00
Fish oil^d^	1.20	1.20	1.20	1.20	1.20
Soy Lecithin	5.00	5.00	5.00	5.00	5.00
Soybean oil^d^	2.00	2.00	2.00	2.00	2.00
$\text{Ca}{\left({\text{H}}_{2}{\text{PO}}_{3}\right)}_{2}^{\text{d}}$	1.00	1.00	1.00	1.00	1.00
Vitamin mixture^e^	1.30	1.30	1.30	1.30	1.30
Mineral mixture^f^	1.00	1.00	1.00	1.00	1.00
Microcrystalline cellulos^e^	2.27	1.70	1.14	0.57	0
Proximate analysis (Mean values, % dry weight)					
Crude protein	59.22	59.31	59.42	59.36	59.30
Crude lipid	13.12	13.04	13.22	13.30	13.18
Ash	16.11	16.34	16.34	16.42	16.73
DH (% crude protein)^g^	11.16	18.04	23.73	28.52	37.57

a The composition of Premixed protein is shown in **Supplementary Table1**;

b Protein hydrolysate is made by Premixed protein;

c Supplied by Weifang Xipu Biotechnology Co., Ltd;

d Supplied by Zhejiang Xinxin Tian’en Aquatic Feed Corporation (Jiaxing, China): wheat gluten meal, crude protein, 80.05%; crude lipid, 1.13%;

e Vitamin Premix (mg/kg diet): vitamin A, 16000 IU; vitamin B1, 17.80; vitamin B2, 48; vitamin B6, 29.52; vitamin B12, 0.24; vitamin C, 800; vitamin D3, 8000 IU; vitamin E, 160; vitamin K3, 14.72; choline chloride, 1500; niacinamide, 79.20; inositol, 320; calcium-pantothenate, 73.60; biotin, 0.64; folic acid, 6.40;

f Mineral Premix (mg/kg diet): I (Ca (IO_3_)_2_), 1.63; Cu (CuSO_4_), 2.00; Fe (FeSO_4_), 21.10; Co (CoCl_2_), 0.24; Zn (ZnSO_4_), 34.4; Se (Na_2_SeO_3_), 0.18; Mn (MnSO_4_), 6.20.

g DH, Degree of hydrolysis.

**Table 2:** Amino acids composition of experimental diets (g/100g diet, dry matter)*.

Amino acids (AA)	Experimental diets				
	PH0	PH25	PH50	PH75	PH100
Essential amino acids (EAAs)					
Threonine	2.41	2.40	2.31	2.46	2.39
Valine	2.65	2.65	2.68	2.63	2.66
Methionine	1.53	1.54	1.55	1.46	1.52
Isoleucine	2.16	2.17	2.15	2.05	2.12
Leucine	4.22	4.26	4.25	4.25	4.25
Phenylalanine	2.33	2.35	2.33	2.37	2.35
Histidine	1.85	1.81	1.93	2.06	1.93
Lysine	3.40	4.08	4.04	4.15	4.09
Arginine	3.20	3.26	3.01	3.20	3.15
Total EAAs	23.75	24.50	24.25	24.62	24.46
Non-essential amino acids (NEAAs)					
Proline	3.02	2.98	2.94	3.05	2.99
Aspartic acid	5.28	5.25	5.25	5.20	5.23
Glutamic acid	9.22	9.45	9.22	9.35	9.34
Serine	2.86	2.85	2.81	2.87	2.84
Glycine	3.68	3.70	3.67	3.78	3.72
Alanine	3.49	3.44	3.48	3.44	3.45
Cystine	0.74	0.66	0.75	0.75	0.72
Tyrosine	2.03	2.06	2.00	2.17	2.08
Total NEAAs	30.32	30.39	30.11	30.61	30.37
Total AAs	54.07	54.89	54.37	55.23	54.83

*Tryptophan was not determined in the present study.

### 2.2 Experimental Procedure

The proposed animal study was reviewed and approved by the Animal Care and Use Committee of Shanghai Ocean University.

Largemouth bass larvae, 7 days post hatching, were obtained from a commercial hatchery (Foshan, China) and then reared in an indoor temperature-controlled recirculating freshwater system (Zhanjiang, China), located in the joint laboratory of Shanghai Ocean University and Guangdong Evergreen Feed Industry Co., Ltd. The larvae were fed with newly hatched artemia for 2 days, and then weaned through alternate feeding with artemia and micro-diet for three days.

After weaning, triplicate groups of one hundred larvae each were randomly collected from the stock and utilized for measuring initial wet weight (9.50 ± 0. 01mg) and body length (9.61 ± 0.01mm) Then, the remaining larvae from the same stock were randomly assigned to 15 fiberglass tanks at a density of 2500 larvae per tank, and each of the fixe experimental diets were randomly allocated to three tanks. The larvae were fed with their allocated diet to apparent satiation four times daily (6:00, 10:00, 14:00, 18:00) for a period of 26 days. During the feeding trial, the feces, residual diet and dead individuals were promptly removed, by aspirating with a siphon tube. The water was recirculated in the system after being filtered with sponges and coral sand, and disinfected by ultraviolet light, and about 10% water was exchanged daily. During the rearing period, the water temperature was 26 ± 1°C, pH was 7.2 ± 0.2, dissolved oxygen was ≥ 6 mg/L, and ammonia nitrogen content was ≤ 0.2 mg/L.

### 2.3 Sample Collection

At the end of the experimental feeding trial, fish were fasted for 24 h and then anesthetized with eugenol before sampling. The number of total fish and deformed fish in each tank were recorded to determine the survival rate and deformity rate, and then the weight of three replicates of 100 individuals per tank were measured to estimate the final body weight. Successively, 50 fish per tank were collected and stored at -20°C for whole body composition analysis, and another 30 fish per tank were used for the measurement of body length. Eventually, an additional 36 fish were collected per each tank for the following analyses: i) the intestine and dorsal muscle samples were separated from 12 fish per tank for gene expression analysis, ii) the stomach and intestine of 12 fish were collected for enzymatic activity analysis, and muscle tissues for amino acid analysis, and iii) another 12 fish were dissected to collect intestinal samples for the analysis of microbiome.

### 2.4 Analytical Methods

The chemical composition analysis of experimental diets and whole fish body were performed according to standard procedures by AOAC (35). Briefly, moisture was measured by drying samples in an oven at 105°C to constant weight. Ash was measured by burning the sample to a constant weight in a muffle furnace at 550°C for 10 h. Crude protein was determined by the Kjeldahl method (N × 6.25) using an automatic Kjelda nitrogen analyzer (Kjeltec 2200, FOSS, Denmark). Crude lipid was measured by the chloroform-methanol extraction method (36). Amino acid composition was determined by an automatic amino acid analyzerb (S-433D, Sykam, Germany) following the acid hydrolytic method of Llames and Fontaine (37).

### 2.5 Digestive Enzyme Activities

The stomach samples were homogenized in ice-cold phosphate buffer (1:9, w/v), and then centrifuged (6000 rpm, 10 min, 4°C) to separate the supernatant for the analysis of pepsin activity, which was then measured using casein as the reaction substrate (38). The intestinal samples were processed in the same way as the stomach samples. The activity of trypsin was evaluated using L-arginine ethyl ester as the reaction substrate (39). The amylase activity was assayed with starch being used as the reaction substrate (40). According to the method of Bier (1946) (41), lipase activity was assayed using the triglyceride as the substrate. The activity of alkaline phosphatase was measured with disodium phenyl phosphate being used as the substrate (42). All of the enzyme activities were expressed as specific activity, and the content of soluble protein was determined by the method of coomassie brilliant blue (43). The measurements of the above digestive enzyme activities were all performed with commercial kits provided by Nanjing Jiancheng Bio-Engineering Institute (China).

### 2.6 RNA Extraction and Real-Time Quantitative Polymerase Chain Reaction

Total RNA of intestinal and muscle samples was extracted using Trizol Reagent (Takara, Japan) and was electrophoresed on a denaturing agarose gel to test the quality. After that, the RNA was reverse transcribed to first-strand cDNA by the Prime Script™ RT kit (Takara, Japan). The expression of following genes were then measured: amino acid transporter and oligopeptide transporter, L-type amino acid transporter 1 (LAT1), L-type amino acid transporter 2 (LAT2), oligopeptide transporter 2 (PepT2), TOR pathway related genes, target of Rapamycin (TOR), serine/threonine kinase 1 (AKT1), ribosomal protein S6 (S6) and eukaryotic initiation factor 4E binding protein 1 (4EBP-1), AAR pathway related genes, eukaryotic initiation factor 2α (eIF2α), activating transcription factor 4 (ATF4) and regulated in development and DNA damage responses 1 (REDD1), and inflammatory related genes, tumor necrosis factor α (TNF-α), interleukin-8 (IL-8), 5-lipoxygenase (5-LOX) transforming growth factor β (TGF-β), interleukin-10 (IL-10), and β-actin was used as house-keeping gene. The primers used in the present study are presented in **Table 3**, which were designed based on previous studies (18, 44, 45). Quantitative real-time PCR (RT-qPCR) was conducted in a quantitative thermal cycler (CFX96, Bio-Rad, CA, USA) with the following procedure: 95°C for 2 min, 40 cycles of 95°C for 10s, 57°C for 10 s, and 72°C for 20 s. At the end of reaction, a melting curve analysis was performed to confirm that the single PCR product presented in those reactions. The comparative CT method (2^-ΔΔCt^ method) was used to calculate the expression of gene (46).

**Table 3:** Sequences of the specific primers used in real-time quantitative PCR.

Gene	Forward sequence (5’-3’)	Reverse sequence (5’-3’)
LAT1	CCAAAGCACGACAGACCTACA	ACCAACCTGGCATATTTCACC
LAT2	GGTGACCACAGGGATAGAGATG	TTGCTTACGGAGGCTGGAACTT
PepT2	TGCACATCCCCTCTCAGTACG	CAAGTCAGTTGGAGCCATTCC
TOR	TCAGGACCTCTTCTCATTGGC	CCTCTCCCACCATGTTTCTCT
AKT1	CACCGTAGAACCGAGCCCGCT	CGCCATGAAGATCCTAAAGAA
S6	GCCAATCTCAGCGTTCTCAAC	CTGCCTAACATCATCCTCCTT
4EBP1	AGCAGGAACTTTCGGTCATA	GTCAATGGGCAGTCAGAAGA
eIF2α	TAAGTCCAGCCCATCCAAAA	CACCCGAGGAGGCCATCAAG
ATF4	GGAGACCAGGAAGATGCGTAG	TGTCCAGCAGCAGTGATGACA
REDD1	TGACCTGTGTCCCTCTAATGA	ATGTGCTCCAGAAGTTTCTCA
TNF-α	CTTCGTCTACAGCCAGGCATCG	TTTGGCCACACCGACCTCACC
IL-8	CGTTGAACAGACTGGGAGAGATG	AGTGGGATGGCTTCATTATCTTGT
5-LOX	GGGATTTTATCGGGGGAC	AACGAGGGAAAGAGGCTG
TGF-β	GCTCAAAGAGAGCGAGGATG	TCCTCTACCATTCGCAATCC
IL-10	CGGCACAGAAATCCCAGAGC	CAGCAGGCTCACAAAATAAACATCT
β-actin	CTCTGGGCAACGGAACCTCT	GTGCGTGACATCAAGGAGAAGC

LAT1, L-type amino acid transporter 1; LAT2 L-type amino acid transporter 2; PepT2, oligopeptide transporter 2; TOR, target of Rapamycin; AKT1, protein kinase B α; S6, ribosomal protein S6; 4EBP-1, eukaryotic initiation factor 4E binding protein 1; eIF2α, eukaryotic initiation factor 2α; ATF4, activating transcription factor 4; REDD1, regulated in development and DNA damage responses 1; TNF-α, tumor necrosis factor α; IL-8, interleukin-8; 5-LOX, 5-lipoxygenase; TGF-β, transforming growth factor β; IL-10, interleukin-10.

### 2.7 DNA Extraction, Amplification, and Sequencing of Gut 16S rRNA Genes

The results of the present study conformed the beneficial roles of dietary protein hydrolysates on elevating growth performance, reducing deformity rate and improving digestive enzyme activity, and most optimal values were observed in the PH100 group (Described in the Result part). Therefore, microbiota analysis was performed only on two of the five treatments, PH0 and PH100, because of logistic and cost limitations. The bacterial DNA of control group and PH100 group were extracted using the E.Z.N.A.^®^ Soil DNA Kit (Omega, USA). The integrity of DNA was evaluated by 1% agarose gel, and the concentration and purity of DNA were determined using a NanoDrop 2000 UV-Vis spectrophotometer (Thermo Scientific, Wilmington, USA). Amplification of the V3-V4 region of bacterial 16S rRNA gene was conducted with PCR thermal cycler with the 338F/806R primer set (ABI, USA). The PCR program was set up as follows: 95°C for 3 min, 27 cycles of 95°C for 30 s, 55°C for 30 s and 72°C for 45 s, and 72°C for 10 min. Then, amplification product was extracted from 2% agarose gels, and purified with the AxyPrep DNA Gel Extraction Kit (Axygen, USA). Subsequently, the purified products were paired-end sequenced using an Illumina MiSeq PE300 platform (Shanghai Bio-Pharm Technology Co. Ltd). The raw reads have then been stored in the NCBI Sequence Read Archive (SRA) database with the accession number PRJNA795583.

### 2.7 Bioinformatics Analysis

The raw fastq files were demultiplexed and quality-filtered using the Quantitative Insights into Microbial Ecology (QIIME) quality filters (47), and then were processed with the FLASH software (48). Sequences were classified into different operational taxonomic units (OTUs) according to the 97% similarity threshold using UPARSE, and then the chimeric sequences were removed using UCHIME Algorithm (49). The phylogenetic affiliation of each 16S rRNA gene sequence was analyzed through the RDP Classifier against the 16S rRNA database (eg. Silva v138), and a confidence threshold set to 70% (50). The alpha diversity analysis, Simpson index and Chao index were calculated with QIIME and displayed with R software. The beta diversity analysis, principal coordinates analysis (PCoA) and non-metric multidimensional scaling (NMDS) ordination were implemented using the same method as above. The intestine bacterial diversity and OTU richness were analyzed by the Welch’s t-test, and the statistical significance was set as *P* < 0.05. The heatmaps were obtained from the results of bacterial clustering at the genus levels. A linear discriminant analysis (LDA) effect size (LEfSe) was performed to identify the different abundant taxa between the PH0 group and PH100 groups.

### 2.8 Parameters and Statistical Analysis

The following parameters were computed using the following formula:

Survival rate (SR, %) = final fish number/initial fish number × 100;

Deformity rate (DR, %) = deformity fish number/final fish number × 100;

Specific growth rate (SGR, %/d) = (Ln (final body weight) - Ln (initial body weight)) ×100/days.

Results were presented as mean ± S.E.M (standard error of the mean). All data were subjected to a one-way analysis of variance (ANOVA) followed using SPSS, followed by the Ducan’s multiple range test, to identify differences across the experimental treatments. However, given the experimental design and to assess the presence of possible trends, polynomial contrasts (linear and quadratic) were used to test the effect of the dietary protein hydrolysates inclusion level on all the variables and parameters measured. When both linear and quadratic significant regressions were recorded, linearity, being the simpler, was selected as the model for describing the observed trend. The intestinal bacterial diversity and OTU richness were implemented only on two treatments only (the two extreme treatments, being PH0, control, and PH100) and were then analyzed by the Welch’s t-test. The level of significance was set at *P* < 0.05.

## 3 Results

### 3.1 Growth Performance

The inclusion of protein hydrolysates linearly increased the final body weight (FBW) and final body length (FBL) of larval largemouth bass compared to the control group (*P* < 0.05) (**Table 4**). The variation of specific growth rate (SGR) followed a similar pattern with that of FBW (*P* < 0.05). The survival rate (SR) was increased in a linear manner with the inclusion of protein hydrolysates (*P* < 0.05). Meanwhile, the inclusion of protein hydrolysates linearly reduced the deformity rate (*P* < 0.05), and the minimum value was observed in the PH100 group (**Table 4**).

**Table 4:** Effects of dietary on growth performance of largemouth bass larvae fed the experimental diets for 26 days.

	Experimental diets					Regression (*P*, R^2^)	
	PH0	PH25	PH50	PH75	PH100	Linear	Quadratic
IBW (mg)	9.50 ± 0.01	9.50 ± 0.01	9.50 ± 0.01	9.50 ± 0.01	9.50 ± 0.01	NA	NA
IBL (mm)	9.61 ± 0.01	9.61 ± 0.01	9.61 ± 0.01	9.61 ± 0.01	9.61 ± 0.01	NA	NA
FBW (g)	1.18 ± 0.09^b^	1.34 ± 0.06^a^	1.37 ± 0.02^a^	1.39 ± 0.13^a^	1.43 ± 0.08^a^	0.002, 0.519	0.005, 0.590
FBL (cm)	3.93 ± 0.09^c^	4.05 ± 0.06^bc^	4.16 ± 0.02^ab^	4.18 ± 0.13^ab^	4.28 ± 0.08^a^	< 0.001, 0.757	< 0.001, 0.766
DR (%)	23.01 ± 1.94^a^	15.01 ± 0.28^b^	14.05 ± 2.01^b^	4.45 ± 1.29^c^	0.48 ± 0.29^c^	< 0.001, 0.905	< 0.001, 0.917
SR (%)	29.27 ± 1.18^c^	32.71 ± 3.63^bc^	39.19 ± 3.30^ab^	42.75 ± 0.48^a^	35.59 ± 1.69^abc^	0.033, 0.304	0.008, 0.550,
SGR (%)	18.56 ± 0.16^b^	19.04 ± 0.10^a^	19.13 ± 0.03^a^	19.17 ± 0.21^a^	19.30 ± 0.12^a^	0.003, 0.509	0.005, 0.588

Values (means ± SEM, N = 3) within a row with a common superscript letter are not significantly different from other dietary treatments (P > 0.05).

NA, not applicable; IBW, initial body weight; IBL, initial body length; FBW, final body weight; DR, deformity rate; SR, survival rate; SGR, specific growth rate.

### 3.2. Body Composition

No statistical difference was observed in whole fish body moisture content among treatments (*P* > 0.05) (**Table 5**). The inclusion of protein hydrolysates resulted in no direct relationship on crude protein content of whole fish body (*P* < 0.05). However, dietary protein hydrolysates decreased crude protein content in PH25, PH75 and PH100 compared to the control group (*P* < 0.05) (**Table 5**). The crude lipid content of whole fish body was increased in a linear manner with the inclusion of protein hydrolysates (*P* < 0.05). A negative linear decrease was observed in the ash content of whole fish body with the increase of dietary protein hydrolysates (*P* < 0.05) (**Table 5**).

**Table 5:** Whole-body composition (% live weight) of largemouth bass larvae fed the experimental diets for 26 days.

	Experimental diets					Regression (P, R^2^)	
	PH0	PH25	PH50	PH75	PH100	Linear	Quadratic
Moisture (%)	77.57 ± 0.33	77.21 ± 0.11	76.87 ± 0.3	77.47 ± 0.62	77.26 ± 0.33	0.763, 0.007	0.664, 0.066
Protein (%)	14.63 ± 0.04^a^	14.19 ± 0.01^c^	14.54 ± 0.01^ab^	14.42 ± 0.08^b^	14.20 ± 0.08^c^	0.123, 0.173	0.317, 0.174
Lipid (%)	3.79 ± 0.11^b^	4.08 ± 0.10^ab^	4.00 ± 0.06^ab^	4.18 ± 0.20^a^	4.09 ± 0.25^ab^	0.015, 0.375	0.017, 0.492
Ash (%)	2.84 ± 0.03^a^	2.82 ± 0.10^a^	2.75 ± 0.01^ab^	2.65 ± 0.05^b^	2.69 ± 0.02^b^	< 0.001, 0.657	0.001, 0.677

Values (means ± SEM, N = 3) within a row with a common superscript letter are not significantly different from other dietary treatments (P > 0.05).

### 3.3 Amino Acids Composition

The amino acids (AAs) profile of larvae muscle is reported in **Table 6**. The inclusion of protein hydrolysates produced a linear decrease of the content of total essential amino acids (EAAs) (*P* < 0.05). However, no individual significant regression was observed in the content of threonine, methionine, phenylalanine and arginine as dietary protein hydrolysates increased (*P* > 0.05) (**Table 6**), but the threonine content in the PH25 group was significantly higher than other treatments (*P* < 0.05). The content of valine, leucine and histidine was decreased linearly with the increase of dietary protein hydrolysates (*P* < 0.05). However, the content of lysine was increased to a plateau in the PH25 group and then decreased, and the trend was well described by a quadratic regression (*P* < 0.05) (**Table 6**). Similarly, the content of total non-essential amino acids and total AAs changed in a quadratic manner, and the maximum value was observed in the PH25 group (*P* < 0.05). The inclusion of protein hydrolysates led to no regression influence on the content of proline, serine, alanine and cystine (*P* > 0.05). However, the proline content in the PH25 group was significantly higher than that in the control group, and the cystine in the PH50 group was significantly higher than that in the control group (*P* < 0.05). The content of aspartic acid was increased to a plateau in the PH50 group and then decreased quadratically (*P* < 0.05). Meanwhile, the inclusion of protein hydrolysates influenced the content of glutamic acid and glycine in a quadratic manner, and the maximum value was observed in the pH25 group (*P* < 0.05). Eventually, the content of tyrosine linearly decreased with the inclusion of protein hydrolysates (*P* < 0.05) (**Table 6**).

**Table 6:** The amino acids composition (% dry weight) in the muscle of largemouth bass fed the experimental diets for 26 days.

	Experimental diets					Regression (*P*, R^2^)	
	PH0	PH25	PH50	PH75	PH100	Linear	Quadratic
Threonine	3.70 ± 0.04^b^	4.09 ± 0.05^a^	3.77 ± 0.08^b^	3.73 ± 0.05^b^	3.66 ± 0.03^b^	0.169, 0.140	0.088, 0.333
Valine	2.99 ± 0.15	3.05 ± 0.03	2.94 ± 0.03	2.92 ± 0.11	2.69 ± 0.02	0.021, 0.348	0.025, 0.458
Methionine	2.63 ± 0.06	2.63 ± 0.07	2.57 ± 0.05	2.57 ± 0.01	2.55 ± 0.03	0.126, 0.107	0.323, 0.172
Isoleucine	2.80 ± 0.17	2.86 ± 0.01	2.65 ± 0.07	2.68 ± 0.04	2.83 ± 0.05	0.664, 0.015	0.441, 0.127
Leucine	6.87 ± 0.20^ab^	7.01 ± 0.06^a^	6.79 ± 0.05^ab^	6.57 ± 0.03^ab^	6.54 ± 0.04^b^	0.005, 0.468	0.015, 0.501
Phenylalanine	3.76 ± 0.40	3.62 ± 0.08	3.51 ± 0.11	3.52 ± 0.07	3.45 ± 0.06	0.205, 0.121	0.426, 0.133
Histidine	3.27 ± 0.06	3.19 ± 0.03	3.18 ± 0.12	3.08 ± 0.07	3.06 ± 0.03	0.017, 0.366	0.064, 0.367
Lysine	6.59 ± 0.08^b^	7.12 ± 0.05^a^	7.02 ± 0.11^a^	6.95 ± 0.06^a^	6.95 ± 0.05^a^	0.159, 0.146	0.016, 0.499
Arginine	8.42 ± 0.21	8.19 ± 0.10	8.20 ± 0.24	8.20 ± 0.13	8.46 ± 0.11	0.826, 0.004	0.293, 0.185
Total EAAs	41.04 ± 0.53	41.75 ± 0.30	40.63 ± 0.46	40.22 ± 0.21	40.19 ± 0.25	0.023, 0.338	0.074, 0.352
Proline	2.25 ± 0.02^b^	2.39 ± 0.01^a^	2.34 ± 0.05^ab^	2.36 ± 0.04^ab^	2.37 ± 0.02^ab^	0.094, 0.201	0.091, 0.330
Aspartic acid	8.69 ± 0.14^ab^	8.86 ± 0.10^ab^	8.98 ± 0.11^a^	8.55 ± 0.03^ab^	8.5 ± 0.07^b^	0.117, 0.178	0.029, 0.446
Glutamic acid	11.23 ± 0.14^c^	12.26 ± 0.12^a^	11.97 ± 0.20^a^	11.87 ± 0.08^ab^	11.49 ± 0.08^bc^	0.877, 0.002	0.004, 0.599
Serine	3.82 ± 0.08	4.00 ± 0.10	3.78 ± 0.15	3.75 ± 0.03	3.71 ± 0.01	0.138, 0.161	0.253, 0.205
Glycine	3.54 ± 0.08^ab^	3.69 ± 0.04^a^	3.58 ± 0.06^ab^	3.55 ± 0.04^ab^	3.39 ± 0.04^b^	0.057, 0.252	0.015, 0.502
Alanine	5.04 ± 0.13	5.20 ± 0.05	5.07 ± 0.05	5.01 ± 0.03	4.92 ± 0.03	0.102, 0.192	0.081, 0.343
Cystine	0.39 ± 0.01^b^	0.46 ± 0.01^ab^	0.52 ± 0.05^a^	0.43 ± 0.01^ab^	0.46 ± 0.01^ab^	0.334, 0.072	0.094, 0.326
Tyrosine	3.05 ± 0.24	3.12 ± 0.05	2.86 ± 0.09	2.72 ± 0.04	2.71 ± 0.01	0.008, 0.428	0.035, 0.429
Total NEAAs	38.00 ± 0.30^b^	39.98 ± 0.39^a^	39.10 ± 0.63^ab^	38.23 ± 0.03^b^	37.51 ± 0.13^b^	0.171, 0.139	0.010, 0.535
Total AAs	79.04 ± 0.82^b^	81.73 ± 0.58^a^	79.72 ± 1.10^ab^	78.46 ± 0.19^b^	77.73 ± 0.12^b^	0.058, 0.250	0.030, 0.441

Values (means ± SEM, N = 3) within a row with a common superscript letter are not significantly different from the other dietary groups (P > 0.05). Tryptophan was not determined in the present study. EAA, essential amino acids; NEAA, non-essential amino acids.

### 3.4 Digestive Enzyme Activities

The inclusion of protein hydrolysates linearly elevated the activity of pepsin, and the maximum value was observed in the PH100 group (*P* < 0.05) (**Table 7**). Meanwhile, the activity of trypsin was significantly increased in a linear manner with the inclusion of protein hydrolysates (*P* < 0.05). No significant regression was observed in the activity of amylase and alkaline phosphatase with the inclusion of protein hydrolysates (*P* > 0.05). However, the activity of amylase in the PH100 group was significantly lower than that in PH0 and PH75 groups (*P* < 0.05). The inclusion of protein hydrolysates elevated the activity of alkaline phosphatase, and its value in the PH50 group was significantly higher than the control group (*P* < 0.05) (**Table 7**).

**Table 7:** The activities of digestive enzymes in the gastrointestinal tract of largemouth bass larvae fed with experimental diets for 26 days.

	Experimental diets					Regression (*P*, R^2^)	
	PH0	PH25	PH50	PH75	PH100	Linear	Quadratic
Pepsin (U/mgprot)	**1.96 ± 0.03b**	2.23 ± 0.05^ab^	2.23 ± 0.02^ab^	2.13 ± 0.04^ab^	2.67 ± 0.10^a^	0.051, 0.262	0.153, 0.269
Trypsin (U/mgprot)	859.76 ± 15.80^c^	1459.54 ± 90.93^b^	1913.52 ± 81.06^a^	1252.55 ± 66.17^b^	1851.92 ± 48.75^a^	0.013, 0.392	0.015, 0.502
AMS (U/gprot)	25.44 ± 1.64^a^	24.23 ± 2.5^ab^	24.64 ± 1.27^ab^	29.22 ± 0.20^a^	16.61 ± 1.92^b^	0.196, 0.125	0.266, 0.198
AKP (U/mgprot)	0.78 ± 0.03^b^	0.79 ± 0.05^b^	1.01 ± 0.02^a^	0.83 ± 0.04^ab^	0.91 ± 0.10^ab^	0.235, 0.107	0.150, 0.271

Values (means ± standard error of the mean, SEM, N = 3) within a row with a common superscript letter are not significantly different from the other dietary groups (P > 0.05). AMS, amylase; AKP, alkaline phosphatase.

### 3.5 Gene Expression Analysis

**Figure 1:**
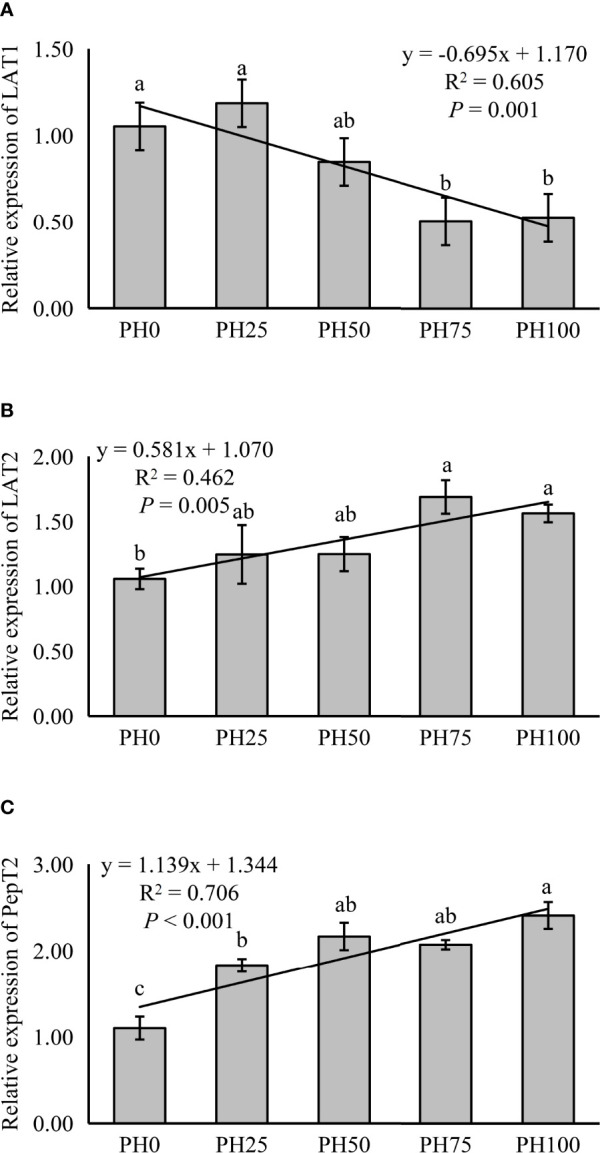
Relative expression of Peptide and AA transporter, L-type amino acid transporter 1 (LAT1) **(A)**, L-type amino acid transporter 2 (LAT2) **(B)**, oligopeptide transporter 2 (PepT2) **(C)**, in intestine of largemouth bass fed the experimental diets for 26 days. Values (means ± standard error of the mean, SEM) in bars that have the same letter are not significantly different (*P* > 0.05; Duncan’s test) among treatments (N = 3). LAT1: *P*_linear_ = 0.001, ${\text{R}}_{\text{linear}}^{2}=0.605$ ; *P*_quadratic_ = 0.003, ${\text{R}}_{\text{quadratic}}^{2}=0.612$ ; LAT2: *P*_linear_ = 0.005, ${\text{R}}_{\text{linear}}^{2}=0.462$ ; *P*_quadratic_ = 0.023, ${\text{R}}_{\text{quadratic}}^{2}=0.467$ ; PepT2: *P*_linear_ < 0.001, ${\text{R}}_{\text{linear}}^{2}=0.706$ ; *P*_quadratic_ < 0.001, ${\text{R}}_{\text{quadratic}}^{2}=0.798$ .

The expression of LAT1 was linearly inhibited in larval intestine with the inclusion of protein hydrolysates (*P* < 0.05) (**Figure 1**). However, the expression of intestinal LAT2 followed an opposite pattern with that of the LAT1, and a positive linear response was observed as protein hydrolysates increased (*P* < 0.05). The supplementation of protein hydrolysates led to a linear increased expression of intestinal PepT2 (*P* < 0.05), with the highest expression in the PH100 group (**Figure 1**).

**Figure 2:**
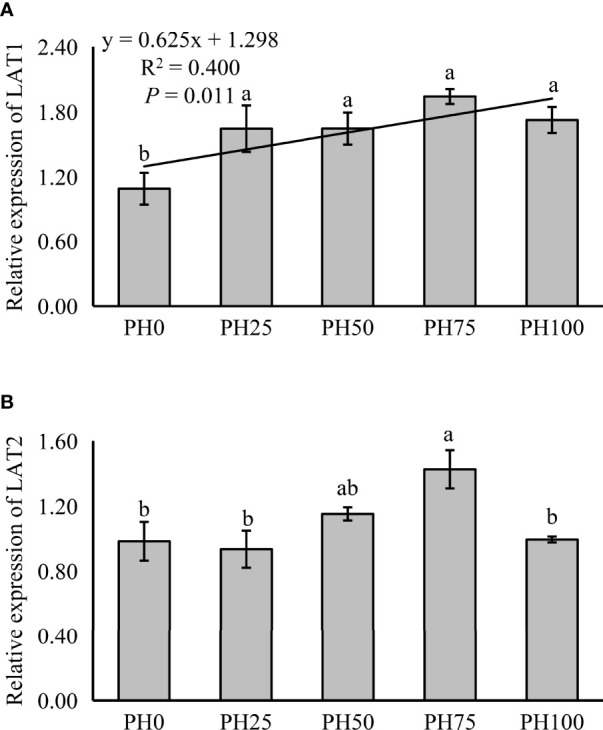
Relative expression of AA transporter, L-type amino acid transporter 1 (LAT1) **(A)**, L-type amino acid transporter 2 (LAT2) **(B)**, in muscle of largemouth bass fed the experimental diets for 26 days. Values (means ± standard error of the mean, SEM) in bars that have the same letter are not significantly different (*P* > 0.05; Duncan’s test) among treatments (N = 3). LAT1: *P*_linear_ = 0.011, ${\text{R}}_{\text{linear}}^{2}=0.400$ ; *P*_quadratic_ = 0.005, ${\text{R}}_{\text{quadratic}}^{2}=0.582$ ; LAT2: *P*_linear_ = 0.235, ${\text{R}}_{\text{linear}}^{2}=0.107$ ; *P*_quadratic_ = 0.175, ${\text{R}}_{\text{quadratic}}^{2}=0.252$ .

The expression of amino acid transporter and oligopeptide transporter, TOR pathway and AAR pathway related genes was also detected in muscle in the present study. The expression of muscle LAT1 was linearly increased with the inclusion of protein hydrolysates (*P* < 0.05) (**Figure 2**). However, the expression of LAT2 was not affected by the inclusion level of protein hydrolysates (*P* > 0.05), but its expression in the PH75 group was significantly higher than other treatments, except the PH50 group (*P* < 0.05) (**Figure 2**).

**Figure 3:**
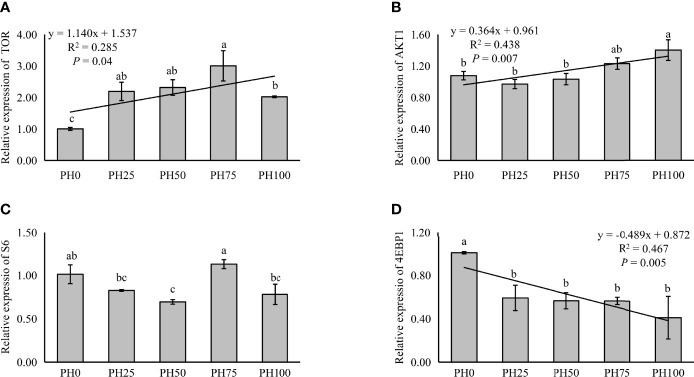
Relative expression of TOR signaling pathway, target of rapamycin (TOR) **(A)**, protein kinase B α (AKT1) **(B)**, ribosomal protein S6 (S6) **(C)**, and eukaryotic initiation factor 4E (eIF4E) binding protein 1 (4EBP1) **(D)**, in muscle of largemouth bass fed the experimental diets for 26 days. Values (means ± standard error of the mean, SEM) in bars that have the same letter are not significantly different (*P* > 0.05; Duncan’s test) among treatments (N = 3). TOR: *P*_linear_ = 0.040, ${\text{R}}_{\text{linear}}^{2}=0.285$ ; *P*_quadratic_ = 0.002, ${\text{R}}_{\text{quadratic}}^{2}=0.664$ ; AKT1: *P*_linear_ = 0.007, ${\text{R}}_{\text{linear}}^{2}=0.438$ ; *P*_quadratic_ = 0.003, ${\text{R}}_{\text{quadratic}}^{2}=0.619$ ; S6: *P*_linear_ = 0.673, ${\text{R}}_{\text{linear}}^{2}=0.014$ ; *P*_quadratic_ = 0.796, ${\text{R}}_{\text{quadratic}}^{2}=0.037$ ; 4EBP1: *P*_linear_ = 0.005, ${\text{R}}_{\text{linear}}^{2}=0.467$ ; *P*_quadratic_ = 0.010, ${\text{R}}_{\text{quadratic}}^{2}=0.536$ .

**Figure 4:**
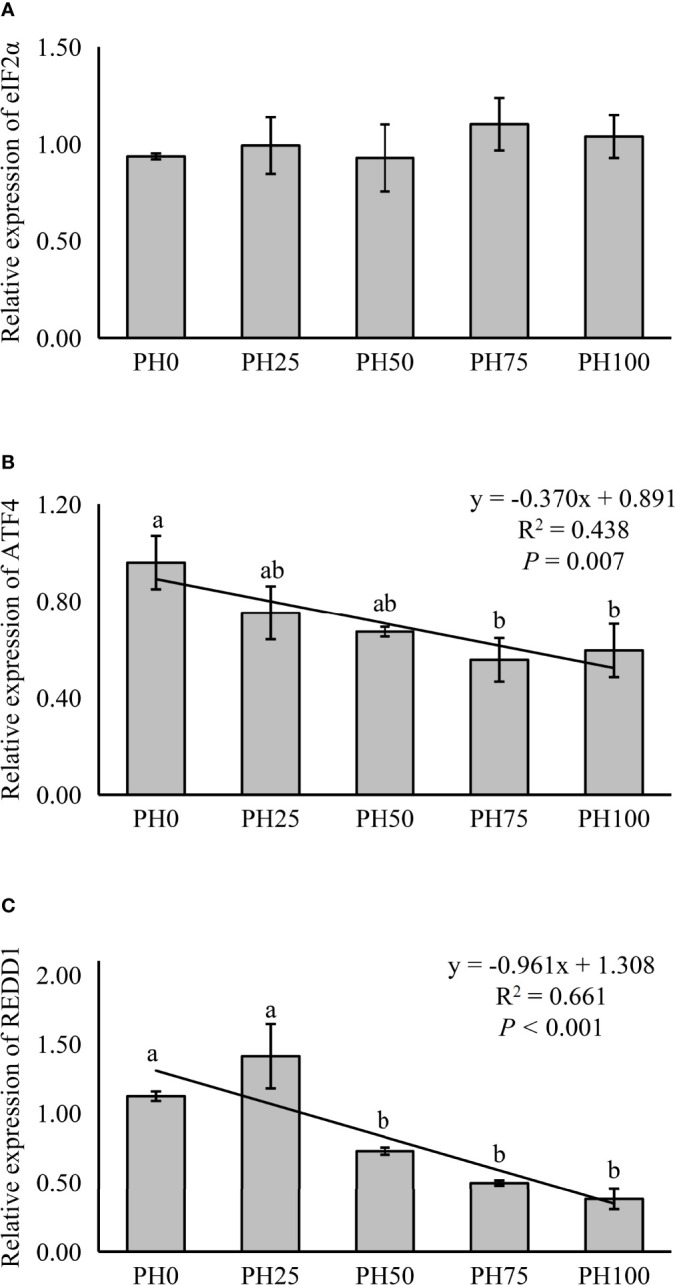
Relative expression of AAR signaling pathway, eukaryotic initiation factor 2α (eIF2α) **(A)**, ATF4, activating transcription factor 4 (ATF4) **(B)**, and regulated in development and DNA damage responses 1 (REDD1) **(C)**, in muscle of largemouth bass fed the experimental diets for 26 days. Values (means ± standard error of the mean, SEM) in bars that have the same letter are not significantly different (*P* > 0.05; Duncan’s test) among treatments (N = 3). eIF2α: *P*_linear_ = 0.408, ${\text{R}}_{\text{linear}}^{2}=0.053$ ; *P*_quadratic_ = 0.720, ${\text{R}}_{\text{quadratic}}^{2}=0.053$ ; ATF4: *P*_linear_ = 0.007, ${\text{R}}_{\text{linear}}^{2}=0.438$ ; *P*_quadratic_ = 0.013, ${\text{R}}_{\text{quadratic}}^{2}=0.514$ ; REDD1: *P*_linear_ < 0.001, ${\text{R}}_{\text{linear}}^{2}=0.661$ ; *P*_quadratic_ = 0.001, ${\text{R}}_{\text{quadratic}}^{2}=0.671$ .

The expression of TOR was elevated in a linear manner with the inclusion of protein hydrolysates (*P* < 0.05), and the variation in the expression of AKT1 followed a similar pattern (*P* < 0.05) (**Figure 3**). No significant regression was observed in the expression of S6 as dietary protein hydrolysates increased, while its expression in the PH75 group was significantly higher than other treatments, except the control group (*P* < 0.05) (**Figure 3**). However, the inclusion of protein hydrolysates linearly reduced the expression of 4EBP1 with the addition of protein hydrolysates (*P* < 0.05) (**Figure 3**). Dietary protein hydrolysates inclusion recorded no linear, nor quadratic relationship with the expression of eIF2α (*P* > 0.05) (**Figure 4**). However, the inclusion of protein hydrolysates linearly decreased the expression of ATF4 and REDD1 (*P* < 0.05) (**Figure 4**).

**Figure 5:**
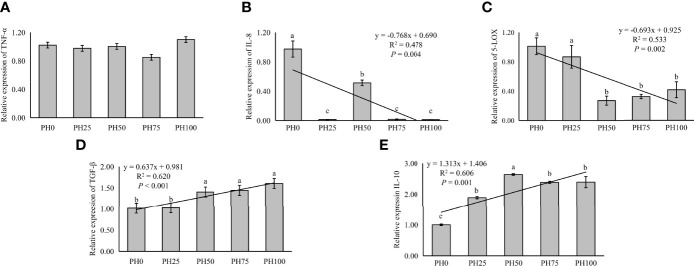
Relative expression of inflammation, tumor necrosis factor α (TNF-α) **(A)**, interleukin-8 (IL-8) **(B)**, and 5-lipoxygenase (5-LOX) **(C)**, and transforming growth factor β (TGF-β) **(D)**, interleukin-10 (IL-10) **(E)**, in intestine of larval largemouth bass fed the experimental diets for 26 days. Values (means ± standard error of the mean, SEM) in bars that have the same letter are not significantly different (*P* > 0.05; Duncan’s test) among treatments (N = 3). TNF-α: *P*_linear_ = 0.704, ${\text{R}}_{\text{linear}}^{2}=0.011$ ; *P*_quadratic_ = 0.406, ${\text{R}}_{\text{quadratic}}^{2}=0.140$ ; IL-8: *P*_linear_ = 0.004, ${\text{R}}_{\text{linear}}^{2}=0.478$ ; *P*_quadratic_ = 0.008, ${\text{R}}_{\text{quadratic}}^{2}=0.555$ ; 5-LOX: *P*_linear_ = 0.002, ${\text{R}}_{\text{linear}}^{2}=0.533$ , *P*_quadratic_ = 0.001, ${\text{R}}_{\text{quadratic}}^{2}=0.693$ ; TGF-β: *P*_linear_ < 0.001, ${\text{R}}_{\text{quadratic}}^{2}=0.620$ ; *P*_quadratic_ = 0.003, ${\text{R}}_{\text{quadratic}}^{2}=0.621$ . IL-10: *P*_linear_ < 0.001, ${\text{R}}_{\text{linear}}^{2}=0.606$ ; *P*_quadratic_ < 0.001, ${\text{R}}_{\text{quadratic}}^{2}=0.914$ .

The expression of inflammation related genes in the intestine was used to evaluate inflammation response of larvae fish to dietary protein hydrolysates. In the present study, no statistical influence was observed in the expression of TNF-α as dietary protein hydrolysates increased (*P* > 0.05) (**Figure 5**). However, the expression of pro-inflammatory cytokine IL-8 was linearly reduced with the inclusion of protein hydrolysates (*P* < 0.05) (**Figure 5**). Meanwhile, the expression of 5-LOX followed a similar pattern with that of IL-8 (*P* < 0.05) (**Figure 5**). However, the supplementation of dietary protein hydrolysates linearly increased the expression of anti-inflammatory cytokine TGF-β (*P* < 0.05) (**Figure 5**). Similarly, the expression of IL-10 was increased in a linear manner with the inclusion of protein hydrolysates (*P* < 0.05) (**Figure 5**).

### 3.6 Microbiota Composition and Diversity Analysis

**Figure 6:**
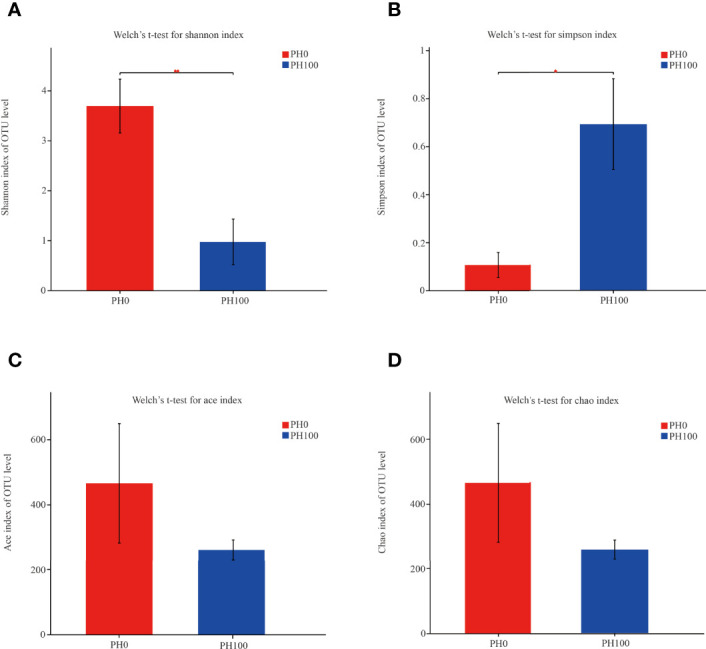
The alpha diversity comparisons analysis, including Shannon diversity index **(A)**, Simpson diversity index **(B)**, Ace species richness index **(C)** and Chao species richness index **(D)** of microbial communities in the intestine of larval largemouth bass between the PH0 and PH100 group. Values (mean ± standard error of the mean, SEM) in bars that have the same letter are not significantly different (*P* > 0.05; Welch’s t-test) between treatments (N = 3).

**Figure 7:**
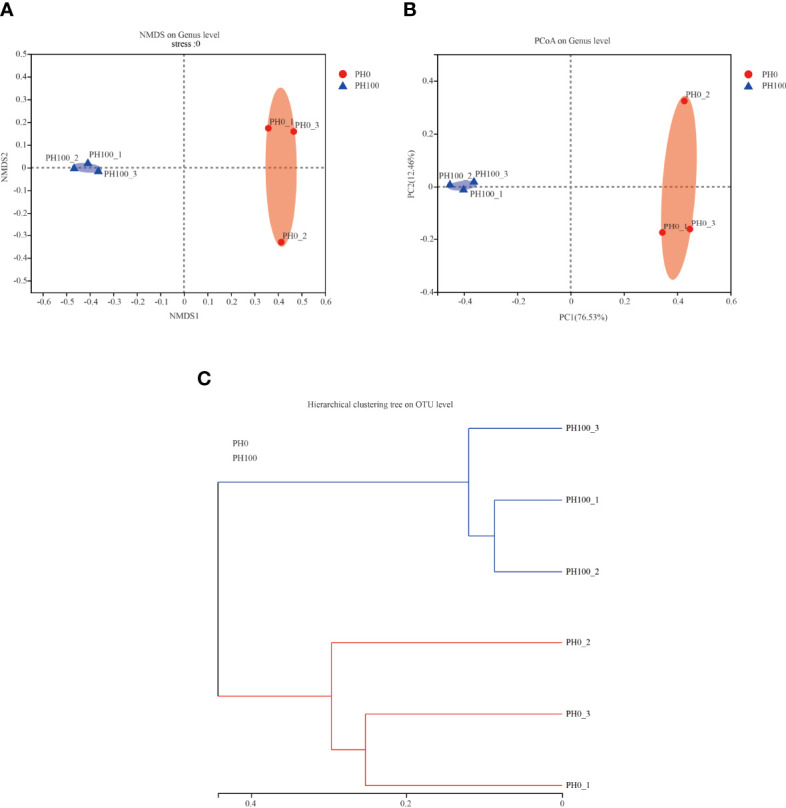
The beta diversity comparisons analysis, including non-metric multidimensional scaling (NMDS) **(A)**, principal component analysis (PCA) **(B)**, and unweighted uniFrac distance matrix **(C)** of microbial communities at genus level in the intestine of larval largemouth bass between the PH0 and PH100 group.

The alpha diversity index, Simpson index of the intestinal microbiota in larvae of the PH100 group was significantly higher than that of the control group (*P* < 0.05), while the variation of the Shannon index followed the opposite variation (*P* < 0.05) (**Figure 6**). However, slightly higher Chao index and Ace index was observed in the PH100 group than the control group (*P* > 0.05) (**Figure 6**). The results of beta diversity comparisons analysis, including non-metric multidimensional scaling (NMDS), principal coordinates analysis (PCoA) and hierarchical clustering tree, showed that the intestine bacterial of PH100 groups was different from the control group (**Figure 7**).

**Figure 8:**
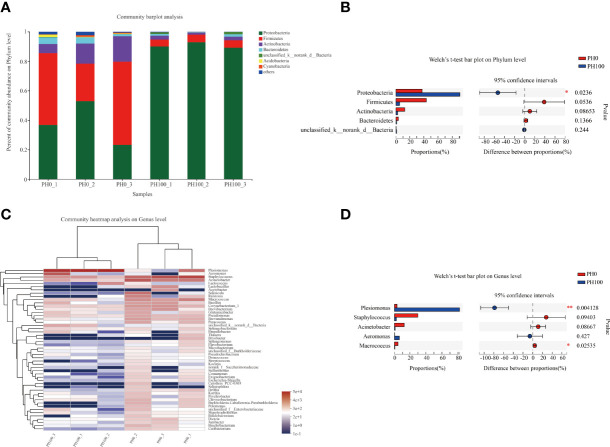
Relative abundances (%) of dominant phyla **(A)** and comparison of five high abundance phyla **(B)** in the intestine of largemouth bass between the PH0 and PH100 group at the phylum level, the phyla with relative abundances lower than 1% were assigned as “others” in Bar map. Heatmap showing the relative abundance of the top 50 most abundant genera in bacterial communities between the PH0 and PH100 group **(C)**. Relative richness of five high abundance genera selected for comparisons in largemouth bass intestine for multiple comparisons among PH0 and PH100 group **(D)**, respectively. * 0.01 < P ≤ 0.05, ** 0.001 < P ≤ 0.01 (Welch’s t-test, N = 3).

The dominant intestine bacterial community of the two groups at the phylum and genus level were showed by the bar map and heatmap, respectively (**Figure 8**). The Proteobacteria, Firmicutes, Bacteroidetes and Actinomycetes were the dominant phyla in the PH0 and PH100 group (**Figure 8**). At the genus level, the *Plesiomonas*, *Staphylococcus*, *Acinetobacter*, *Aeromonas*, and *Macrococcus* were the predominant bacterial in the two groups (**Figure 8**). The Welch’s-test revealed that protein hydrolysates supplementation significantly increased the relative abundance of *Plesiomonas*, while significantly decreased the abundance of the *Staphylococcus*, *Acinetobacter*, *Macrococcus*, *Pseudomonas*, and *Brevundimonas* (*P* < 0.05) (**Figure 8**).

The Venn diagram analysis showed that 20 shared microbial phyla were identified between the control group and PH100 group, and meanwhile, 10 and 1 unique bacterial phyla were detected in the control and PH100 groups, respectively (**Figure 9.1**). At genus level, 223 shared microbial genera were identified between the control group and PH100 group, and 337 and 27 unique bacterial phyla were detected in the control and PH100 groups, respectively (**Figure 9.2**). The most abundant genera in the shared genera (>5%) were *Plesiomonas* (47.50%), *Staphylococcus* (14.68%), and *Acinetobacter* (6.54%) (**Figure 9.2**). The unique intestine microbial genus (>5%) in the larvae of the PH0 treatment was *Acetobacter* (12.46%) and *Bryobacter* (5.45%) (**Figure 9.2**), and the unique genus (>5%) in the larvae of PH100 group was *Atopostipes* (8.55%), *Sanguibacter* (8.55%), *Aequorivita* (7.69%) and *Prevotella* (5.13%) (**Figure 9.2**).

**Figure 9:**
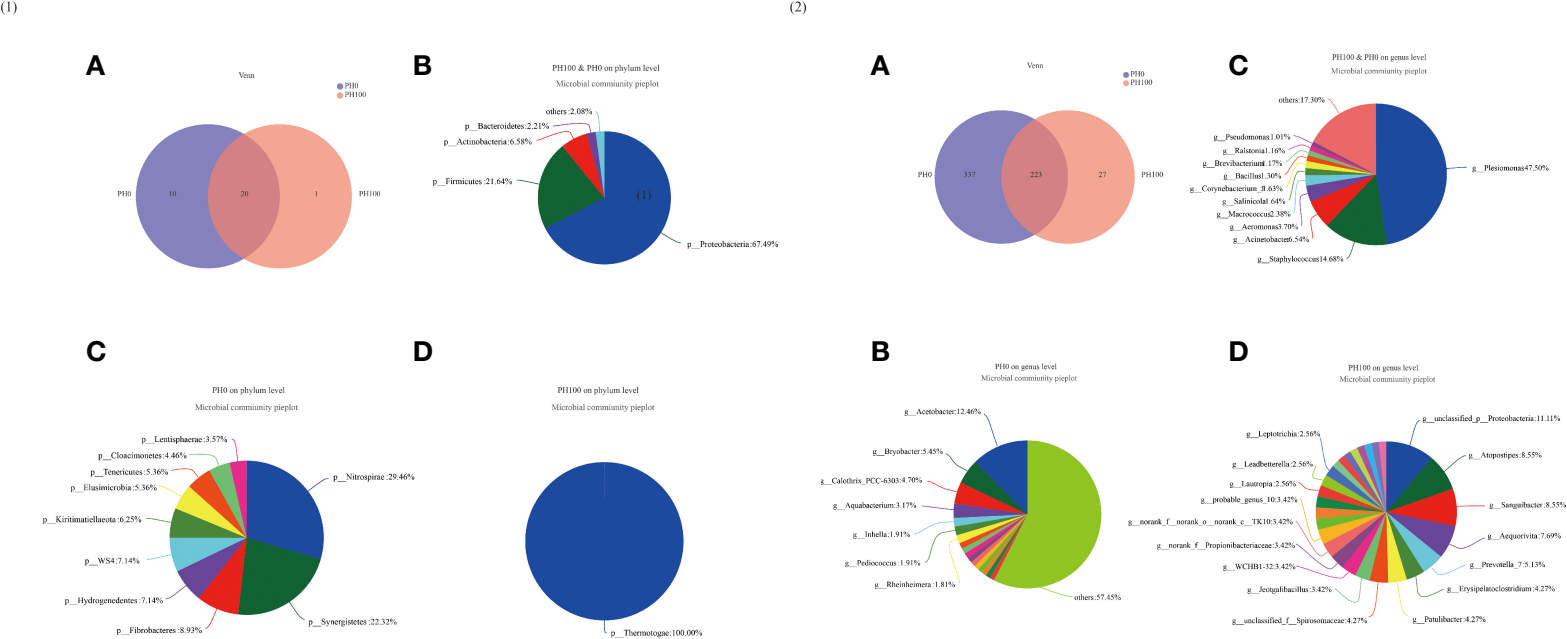
Venn diagram analysis of microbial communities in the intestine of larval largemouth bass between the PH0 and PH100 group. The number **(A)** and community bacterial phylum **(B)** and unique **(C, D)** of overlapping in the intestine of largemouth bass were identified (**Figure 9.1**). The number **(A)** and community bacterial genera **(B)** and unique **(C, D)** of overlapping in the intestine of largemouth bass were identified (**Figure 9.2**).

**Figure 10:**
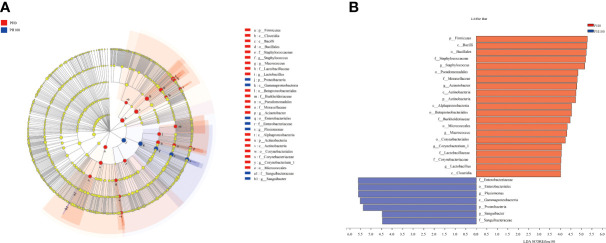
Cladogram showing the phylogenetic distribution of the bacterial lineages associated with dietary protein hydrolysates inclusion. Taxonomic representation of statistically and biologically consistent differences between intestinal microbiota of largemouth bass between the PH0 and PH100 group **(A)**. Differences were represented by the color of the most abundant class (red indicates control group; blue indicates PH100 group). Histogram of linear discriminant analysis (LDA) scores for differentially abundant taxon **(B)**. For interpretation of the references to color in this figure legend, the reader is referred to the web version of this article.

The analysis of intestine microbial community data from the domain to the genus level was evaluated by LEfSe, and there were significant differences in the taxonomic distribution of gut microbial communities between the control group and PH100 group (**Figure 10**). The intestine of larvae fed the diet with protein hydrolysates supplementation exhibited significant enrichment for genus *Plesiomonas*, phylum Proteobacteria, *Sanguibacter* (from family to genus), *Enterobacteriaceae* (from class to family), while the control diet without protein hydrolysates had a higher relative abundance of *Staphylococcus*, *Macrococcus* (from phylum to genus), *Lactobacillus* (from family to genus), *Acinetobacter*, *Corynebacterium_1* (from order to genus) (**Figure 10**).

## 4 Discussion

The experimental *in vivo* feeding trial presented in this study was successfully implemented, and it confirmed the beneficial roles of protein hydrolysates in improving growth performance and survival rates, and reducing deformity rate of largemouth bass larvae, as previously observed also for other finfish species, such as European sea bass (*Dicentrarchus labrax*), Atlantic cod (*Gadus morhua*), and Nile tilapia (*Oreochromis niloticus*) (4, 51, 52). The enzymolysis of intact protein used in the present study produced free amino acids and small peptides with short chain length, which have been reported to act as attractants, and therefore have been suggested to contribute to the elevated feed intake and growth performance in teleosts (53). Meanwhile, the digestion and absorption of the peptides with smaller molecules compared to intact proteins was facilitated in the immature digestive tract of fish larvae (54; 55; 56), and this may have also accounted for the improved growth performance of larvae fed the diet with high content of protein hydrolysates. Additionally, the efficiency of abundant dietary peptides has been shown to contribute in the maintenance of a harmonious development of larvae, especially the skeletal development (19), and this may have accounted for the decreased deformity rate in the present study.

It is known that, at early stages of development of larval fish, the stomach is not fully developed, and only later, with the development of the stomach, pepsin activity is enhanced (57, 58). Therefore, the increased activity of pepsin recorded in larvae receiving protein hydrolysates might suggest that the provision of dietary protein hydrolysates plays a role in a more rapid development of stomach in the larvae. Additionally, the protein hydrolysates inclusion significantly elevated also the activity of trypsin. This phenomenon was also observed in gilthead sea bream (*gilthead seabream*) larvae (59) and in larval large yellow croaker (*Larimichthys crocea*) (7), suggesting that not only the stomach development, but the development of the entire digestive system has been positively affected by the dietary inclusion of protein hydrolysates. In parallel, it is well known that in larval fish amylase activity decreases with the development of pancreatic exocrine secretion (60). Accordingly, the reduced amylase activity observed in fish larvae under the PH100 treatment indicated a likely anticipated development of pancreatic exocrine secretion, compared to larvae fed the control diet. On the other hand, the alkaline phosphatase activity, which is mainly secreted by the intestinal epithelial tissue, and has been suggested to reflect the degree of intestinal development (61), was also elevated with the provision of dietary protein hydrolysates. Nevertheless, in summary it can be reported that the observation of the variations in digestive enzyme activities confirmed that dietary protein hydrolysates played a substantial role in improving the digestive tract development of larval largemouth bass. However, small peptides have been suggested to be absorbed more rapidly compared to larger peptides in some teleosts, including Atlantic halibut (*Hippoglossus hippoglossus*) (62), and Senegalese sole (*Solea senegalensis*) (63), and this might lead to greater utilization of these shorter peptides for catabolic processes and reduced retention. Therefore, the increased small peptides caused by the replacement of un-hydrolyzed protein pre-mixture may partly accounted for the reduced crude protein content of whole fish body in the present study. During the digestive process, dietary intact proteins are broken down into free amino acids and small peptides in the intestinal lumen, which can then be absorbed by peptide and AA transporters (9, 64). Amongst these transporters, the main function of L-type amino acid transporter LAT1 is to facilitate the uptake of larger branched-chain amino acids and aromatic neutral amino acids, such as leucine, isoleucine, valine (65), whereas, the L-type amino acid transporter LAT2 has a broader substrate specificity, as it can transport all neutral amino acids, except proline (66, 67). PepT2 is a further important transporter, and it is considered the main peptide transporter in the intestine (68). In the present study, the inclusion of protein hydrolysates increased the expression of PepT2, and this was consistent with the results observed in common carp (*Cyprinus carpio*) (69). Similarly, the inclusion of protein hydrolysates increased the expression of LAT2, which was partly caused by the increased influx of free AAs, and a similar observation was made in turbot (*Scophthalmus maximus* L.) fed high levels of fish protein hydrolysates, where significantly increased expression of y^+^LAT2 gene in middle and distal intestine was recorded (70). Unexpectedly, however, in the present study the provision of protein hydrolysates significantly down-regulated the expression of LAT1 in intestinal tissues, but elevated its expression in muscle. Previous studies have found that increased availability of essential amino acids increased LAT1 expression in human skeletal muscle (71), but little relevant information is available for fish. At this point, any speculation relative to the possible underlying mechanisms, or the physiological meaning of such reduced LAT1 in the intestine tissues would be unsubstantiated, and it is suggested that this phenomenon, and in particular the divergent trends observed for PepT2, LAT2 and LAT 1 would be further investigated.

TOR and AAR signaling pathways are two distinct but complementary signaling pathways for cellular nutrient sensing, and their main functions are to regulate protein synthesis and downstream metabolism (10). The TOR signaling pathway has been well demonstrated to be involved in the regulation of protein synthesis in mammals (72), and is receiving increasing attention in teleosts too. In the present study, the inclusion of protein hydrolysates elevated the expression of TOR, a result in agreement with some previous studies in fish, such as turbot (70), largemouth bass (18) and black sea bream (*Acanthopagrus schlegelii*) (73). In teleosts, the activation of TOR pathway was observed to be positively correlated with growth performance in Jian carp (*Cyprinus carpio* var. Jian) and tilapia (*Oreochromis niloticus*) (74–76). The eukaryotic initiation factor 4E (elF4E) binding protein 1 (4EBP1) and S6 kinase 1 (S6K1) are the main downstream targets of TOR (11). In mammals, the activation of TOR pathway commonly induces the phosphorylation activation of S6K1 and phosphorylation inhibition 4EBP1 to stimulate translation initiation of protein (77). In the present study, although the expression of S6 was only elevated in the PH75 group, the inclusion of protein hydrolysates significantly decreased the expression of 4EBP1, which was consistent with a previous study in juvenile largemouth bass (18). Additionally, the integration of TOR and AKT1, which is also involved in the regulation of growth and metabolism at multiple levels (78), has been studied and reported in mammals. In the present study, an increased expression of AKT1 was observed in the larvae fed diets with protein hydrolysates inclusion, which is in agreement with previous studies in turbot (70), largemouth bass (18) and black sea bream (73).

Opposite to the TOR pathway, the AAR pathway has a modulatory effect as a suppressor of protein synthesis. An imbalanced amino acids provision has been shown to cause the activation of AAR pathway, which consist of the phosphorylation of eIF2α to suppress protein synthesis, and the activation of the expression of ATF4 to adapt to dietary stress (79, 80). Previous studies in teleosts have confirmed the activation of AAR pathway by an imbalanced dietary amino acids supply (16, 17, 44), and it has also been shown that the AAR pathway can be regulated at the transcriptional levels (34; 18). In the present study, although the expression of eIF2α was not influenced, the expression of ATF4 was significantly decreased by the inclusion of protein hydrolysates, indicating an inhibitory action of protein hydrolysates on the AAR pathway in larval largemouth bass. ATF4 has been demonstrated to induce the expression of REDD1, which, in mammals, has been reported to acts as a negative regulator of the TOR signaling pathway (81). Consistently, a reduced expression of REDD1 caused by protein hydrolysates was observed in the present study, with similar result previously reported in juvenile largemouth bass (18). Therefore, the activation of the TOR pathway and the simultaneous inhibition of the AAR pathway are likely key contributors to the observed improved growth performance of larvae fed diets with protein hydrolysates.

Previous evidence showed that certain level of protein hydrolysates in the diets of fish larvae provides improvements of immune responses (26, 27, 82). Different cytokines are classified as either pro-inflammatory or anti-inflammatory, and play a vital role in the innate defense of fish (83). In the present study, the expression of pro-inflammatory cytokines, 5-LOX and IL-8 was significantly downregulated with the supplementation of protein hydrolysates, indicating a direct inhibitory effect of inflammation responses. In a previous study, it has been shown that histidine inhibited IL-8 at transcriptional level in intestinal epithelial cells (84). In the present study, the relative high content of free histidine content derived from the inclusion of protein hydrolysates may therefore partly account for the depressed IL-8 expression. An additional consideration arises from the well demonstrated effect that anti-inflammatory cytokines, such as TGF-β, have on the inhibition of the production of pro-inflammatory cytokines, thereby suppressing the inflammatory response in teleost (85). In fact, a diametral opposite variation between the expression of anti-inflammatory and pro-inflammatory cytokines has been reported in some teleosts (86–88). Consistently, in the present study, the expression of anti-inflammatory cytokines TGF-β and IL-10 was significantly increased with the inclusion of protein hydrolysates. A possible driver for this observed increased expression of IL-10 could be linked to the increasingly higher content of free glycine in groups with higher protein hydrolysates inclusion, and glycine has been reported, in mammals, to possesses the ability to promote IL-10 expression and consequently reduce inflammatory response (89).

The diversity and abundance of intestinal microbiota has been well demonstrated to affect the host biological processes including digestion and mucosal system development (21, 90), and several factors, such as dietary nutrient composition, have been shown to significantly affect the microbial community composition of fish larvae (91). In the present study, the Proteobacteria, Firmicutes, Actinomycetes and Bacteroidetes, were the dominant phyla in the PH0 and PH100 treatments, which was partly consistent with previous results in other teleosts such as gibel carp (*Carassius auratus gibelio*) (92) and rainbow trout (*Oncorhynchus mykiss*) (93). However, dietary protein hydrolysates supplementation significantly reduced the diversity of the intestine microbial community, which was in line with previous studies in largemouth bass and gilthead seabream (94; 95). Previous studies have revealed that *Plesiomonas* is a normal inhabitant of the fish gut (96), and this genus was also the most dominant microbes observed in African Catfish (97). The antimicrobial effect of *Plesiomonas* has been well clarified (98), and therefore, the decreased inflammatory response of larvae in the PH100 group may have been also partly due to the increased relative abundance of *Plesiomonas*. Another interesting observation is that, in zebrafish, *Staphylococcus aureus*, a member of the genus *Staphylococcus*, have been reported to induce an increased inflammatory response (99). In this study, dietary protein hydrolysates supplementation clearly reduced the relative abundance of *Staphylococcus*, and thus, dietary protein hydrolysates inclusion, by contributing to the regulation of the composition of intestinal microbiota, positively contributed to a reduced intestinal inflammatory response.

In conclusion, the dietary supplementation of protein hydrolysates significantly improved the growth performance, development and health status of largemouth bass larvae. These effects appeared to be mediated by a cascade of effects, starting from an increased palatability of diets containing protein hydrolysates, and then being followed by a more rapid and effective absorption of nutrients, in particular amino acids, which then stimulated protein synthesis *via* the activation of TOR pathway and inhibition of AAR pathway. The increased protein synthesis facilitated gastrointestinal development, further facilitating nutrient absorption, and also modulating the microbiota, which showed reduced presence of the inflammatory *Staphylococcus* and increased presence of antimicrobial *Plesiomonas*. These changes in microbiota, coupled with higher availability of free histidine and free glycine originating from the protein hydrolysates, resulted in increased anti-inflammatory cytokines and reduced pro-inflammatory cytokines, benefitting the overall immune status of fish larvae. Therefore, protein hydrolysates are suggested to be an effective and viable raw material as the main protein sources in diets for largemouth bass larvae in order to ensure the improved overall performance and fish health.

## Data Availability Statement

The datasets presented in this study can be found in online repositories. The names of the repository/repositories and accession number(s) can be found in the article/supplementary material.

## Ethics Statement

The animal study was reviewed and approved by Animal Care and Use Committee of Shanghai Ocean University.

## Author Contributions

ZS: investigation, formal analysis, and writing - original draft; GT: methodology and writing - review and editing; JX: investigation, methodology, and data curation; ZF: investigation, methodology, and data curation; NC: conceptualization, project administration, and funding acquisition; RX and HZ: investigation; SL: conceptualization, supervision, writing - review and editing, and funding acquisition. All authors contributed to the article and approved the submitted version.

## Funding

This work was financially supported by National Key R&D Program of China (2019YFD0900203), China Agriculture Research System of MOF and MARA (CARS-46), National Natural Science Foundation of China (31802308), and Shanghai Talent Development Fund (2019097).

## Conflict of Interest

The authors declare that the research was conducted in the absence of any commercial or financial relationships that could be construed as a potential conflict of interest.

## Publisher’s Note

All claims expressed in this article are solely those of the authors and do not necessarily represent those of their affiliated organizations, or those of the publisher, the editors and the reviewers. Any product that may be evaluated in this article, or claim that may be made by its manufacturer, is not guaranteed or endorsed by the publisher.
