# Supplementary material for: Functional Properties of Protein Hydrolysates on Growth, Digestive Enzyme Activities, Protein Metabolism, and Intestinal Health of Larval Largemouth Bass (Micropterus salmoides)
Source: Front Immunol. 2022 Jul 19;13:913024. doi: 10.3389/fimmu.2022.913024 (PMC9343713; doi:10.3389/fimmu.2022.913024)
Supplement: Supplementary file 1 [file Table_1.docx]

**Table 1**. Formulation and chemical composition of premixed protein (% dry matter).

| Ingredients | level |
| --- | --- |
| White fish meal^a^ | 60 |
| Fermented soybean meal^a^ | 6 |
| Shrimp meal^a^ | 5 |
| Blood meal^a^ | 2 |
| Proximate analysis (Mean values, % dry weight) | |
| Crude protein | 68.21 |
| Crude lipid | 6.69 |
| DH | 40.94 |

^a^Supplied by Zhejiang Xinxin Tian’en Aquatic Feed Corporation (Jiaxing, China)

**Table 2**. Protein hydrolysates peptide molecular weight (Da) distribution of the soluble protein fraction (% total protein)*.

| Peptide molecular weight (Da) | % |
| --- | --- |
| >10000 Da | 0.28 |
| 10000~5000 Da | 1.04 |
| 5000~3000 Da | 2.53 |
| 3000~2000 Da | 4.20 |
| 2000~1000 Da | 14.44 |
| 1000~500 Da | 24.59 |
| 500~180 Da | 36.56 |
| ＜180 Da | 16.37 |

^⁎^ Analysed from Analysis and Testing Center of Jiangnan University (Wuxi, China).

**Table 3.** Free amino acid content of the protein hydrolysates (g/100g, dry matter)*.

| Amino acids (AA) | Protein hydrolysates |
| --- | --- |
| Essential amino acid (EAA) | |
| Threonine | 0.08 |
| Valine | 0.23 |
| Methionine | 0.27 |
| Isoleucine | 0.03 |
| Leucine | 0.13 |
| Phenylalanine | 0.06 |
| Histidine | 0.66 |
| Lysine | 0.18 |
| Arginine | 0.12 |
| Total EAAs | 1.76 |
| Proline | 0.25 |
| Aspartic acid | 0.01 |
| Glutamic acid | 0.19 |
| Serine | 0.07 |
| Glycine | 0.16 |
| Alanine | 0.22 |
| Cystine | 0.04 |
| Tyrosine | 0.07 |
| Total NEAAs | 1.01 |
| Total AAs | 2.77 |

*Tryptophan was not determined in the present study; Analysed from Xi'an United Nations Quality Detection Technology CO., Ltd.
